# Long-Term Fertilization Modifies the Structures of Soil Fulvic Acids and Their Binding Capability with Al

**DOI:** 10.1371/journal.pone.0105567

**Published:** 2014-08-19

**Authors:** Jun Wu, Minjie Wu, Chunping Li, Guanghui Yu

**Affiliations:** 1 Key Laboratory of Soil Environment and Pollution Remediation, Institute of Soil Science, Chinese Academy of Sciences, Nanjing, Jiangsu, PR China; 2 State Key Laboratory of Soil Sustainable Agriculture, Institute of Soil Science, Chinese Academy of Sciences, Nanjing, Jiangsu, PR China; 3 National Engineering Research Center for Organic-based Fertilizers, College of Resources and Environmental Sciences, Nanjing Agricultural University, Nanjing, PR China; Tennessee State University, United States of America

## Abstract

The binding characteristics of organic ligands and minerals in fulvic acids (FAs) with Al are essential for understanding soil C sequestration, remain poorly understood. In this study, Fourier transform infrared (FTIR) spectroscopy combined with two-dimensional correlation spectroscopy (2DCOS) analysis was applied for the first time to explore the binding of Al with organic ligands and minerals in soil FAs. For these analyses, two contrasting treatments were selected from a long-term (i.e., 22-year) fertilization experiment: chemical (NPK) fertilization and swine manure (SM) fertilization. The results showed that the long-term application of organic and inorganic fertilizers to soils had little effect on the compositions of the fluorescent substances and organic ligands in the soil FAs. However, long-term SM fertilization increased the weathered Al and Si concentrations in the soil FAs compared with long-term chemical fertilization. Furthermore, organic ligands in the soil FAs were mainly bound with Al in the NPK treatment, whereas both organic ligands and minerals (Al-O-Si, Si-O) were bound with Al under the M fertilization conditions. Both transmission electron microscopy (TEM) images and X-ray diffraction spectra demonstrated that amorphous and short-range-ordered nanominerals were abundant in the soil FAs from the SM plot in contrast to the soil FAs from the NPK plot. This result illustrates the role nanominerals play in the preservation of soil FAs by during long-term organic fertilization. In summary, the combination of FTIR and 2D correlation spectroscopy is a promising approach for the characterization of the binding capability between soil FAs and Al, and a better understanding FA-Al binding capability will greatly contribute to global C cycling.

## Introduction

Humic substances (HSs), the dominant component of soil organic matter (SOM), are formed by the decomposition of plant, animal, and microbial materials. They are complex, heterogeneous, organic compounds that possess different structures and solubilities and a wide range of molecular weights [1]–[3]. HSs are sequestered in the soil systems and contribute to increases in soil fertility and reductions in greenhouse gas emissions following complexation with metals. HSs are categorized into the following three groups on the basis of their solubility: humic acids (HAs), fulvic acids (FAs), and humins. Among these groups, FAs have the lowest -molecular -weight, with molecular weights ranging from approximately 500 to 2000 Da, and they contain the highest oxygen content in their complex humic groups [4]. Additionally, FAs are the most mobile components among these three groups. Large specific surface areas and strong structural binding sites make FAs potentially important as carrying agents with regard to the binding and bioavailability of metals [5], [6]. Recently, studies have demonstrated that nanominerals are abundant in soil FAs, which affect the nanominerals’ binding behavior with metals [7], [8]. However, the binding behavior between nanominerals in soil FAs and metals remains unclear.

Fourier transform infrared (FTIR) spectroscopy is a commonly used technique that can distinguish between the principal organic ligands and minerals that are found in soil FAs, such as carbohydrates, lignin, cellulose, lipids, proteins, and compounds containing Al-O bonds, through the vibration characteristics of their structural chemical bonds [8], [9]. By determining the FTIR spectra of FAs after a series of metal titrations, it is possible to explore the complexes between the organic ligands in FAs and metals. However, individual FTIR spectral features often overlap [8], [10], complicating investigations of binding between the organic ligands in FAs and metals. Recently, two-dimensional correlation spectroscopy (2DCOS) analysis, which can solve the overlapping peaks problem by distributing the spectral intensity trends within a data set over a second dimension [9]–[11], has been applied to characterize the binding of different organic ligands with metals [8], [12]. It is expected that FTIR spectroscopy combined with 2DCOS analysis can facilitate investigations of the binding of organic ligands in soil FAs with Al at the molecular level.

Fertilization practices have been shown to affect the levels and the quality of SOM within cropping systems [8], [13]–[15]. However, little is known about the binding of organic ligands and minerals in soil FAs with Al. By understanding the binding between organic ligands in soil FAs and Al, the stability and bioavailability of SOM under different fertilization conditions can be better understood. The objectives of this study were 1) to investigate the binding of organic ligands in soil FAs with Al and 2) to explore the mechanism by which SOM is sequestered under different fertilization practices. For these purposes, the following two contrasting treatments were selected from a long-term (i.e., 22-year) fertilization experiment: chemical (NPK) fertilization and swine manure (SM) fertilization.

## Materials and Methods

### Site and soil sample collection

Soils were collected from two contrasting treatments, NPK and SM, at a long-term fertilization experiment station. The long-term fertilization experiment was initiated in September 1990 in fields that were double cropped with wheat and corn at an experiment station of the Chinese Academy of Agricultural Sciences, Qiyang (26°45′ N, 111°52′ E, 120 m altitude), Hunan Province, Southern China. No specific field permits were required for this study. The land accessed is not privately owned or protected. No protected species were sampled. The red soil was classified as Ferralic Cambisol. Concentration of allophane in soils from NPK and SM treatments in 2012 was 0.25±0.05 mg/kg and 1.80±0.13 mg/kg, respectively. A detailed description of the long-term fertilization experiment site can be found elsewhere [8], [16]. In brief, the experimental area receives 1,255 mm of annual precipitation, approximately 70–80% of which occurs from April to October. Mean annual temperature, annual evaporation, annual frost-free days, and sunshine hours are 18°C, 1,470 mm, 300 d, and 1,610 h, respectively. Prior to the experiment, the field had been under an annual wheat-corn rotation for 3 yrs to give soil uniform fertility. The chemical fertilizers used were urea for N, superphosphate for P, and potassium chloride (KCl) for K. The total N applied in the NPK and SM treatments per year is 300 kg/ha.

Soil samples at depths of 0–20 cm were collected in October 2012 using a 5-cm internal diameter auger. Each sample was a composite of ten random cores that were collected from two replicate plots. The fresh soil was mixed thoroughly, air-dried, roots removed, and then sieved through a 2.0-mm sieve for further analysis.

### Extraction and purification of fulvic acids (FAs)

The FA fraction was extracted from the surface soil (0–20 cm) samples following the International Humic Substance Society (IHSS) procedure [17]. The sample was equilibrated to a pH value between 1 and 2 with 1 M HCl at room temperature. The solution volume was adjusted with 0.1 M HCl to provide a final concentration ratio of 10 mL liquid per g dry sample. The suspension was shaken for 1 h, and the supernatant was separated from the residue by low speed centrifugation (i.e., 2000 *g*). The residues were then neutralized with 1 M NaOH. A 0.1 M solution of NaOH was then added to the residues under nitrogen gas using an extractant to sample ratio of 10:1. The suspension was extracted under nitrogen gas with intermittent shaking for a minimum of 4 h. After centrifugation, the supernatant solution was collected and acidified to pH 1.0 with 6 M HCl and was allowed to settle for 12 h before centrifuging again [17]. The FA fraction was concentrated and purified by flowing the FAs through an XAD-8 adsorption resin column (AMBERLITE), and the 0.1 M NaOH eluate was passed through a H^+^-saturated cation exchange resin column (CER, Dowex MAC-3, Sigma) (Hiradate, 2006). The purified FA samples were stored at 4°C until further analysis.

### Fluorescence excitation-emission matrix (EEM) determination

The fluorescence EEMs were measured on a Varian Eclipse fluorescence spectrophotometer in scan mode. Scanning emission (Em) spectra from 250 to 600 nm were obtained in 2 nm increments by varying the excitation (Ex) wavelength from 200 to 500 nm in 10 nm increments [18]. The spectra were recorded at a scan rate of 1200 nm/min using excitation and emission slit band widths of 5 nm. Several preprocessing steps were used to minimize the influence of scatter lines and other attributes of the EEM landscape. The EEM of a control Milli-Q water was subtracted from each sample, and other Rayleigh and Raman scatters were removed by the protocol of Bahram et al. [19]. After that, the EEMs were normalized by dividing the spectra by the corresponding DOC concentrations.

### Al titration test

Aliquots of 25 mL of the diluted solution of FAs were titrated into 40 mL brown, sealed vials containing 0.01 mol/L AlCl_3_ using an automatic syringe. The Al concentrations in the final solutions ranged from 0 to 70 µmol/L. No more than 25 µL of the metal titrant was added during titration to maintain constant pH before and after titration. All titrated solutions were shaken for 24 h at 25°C to ensure complexation equilibrium [20]–[22]. Finally, after freeze-drying the solutions, they were analyzed by FTIR spectroscopy.

### FTIR spectroscopy determination and analysis of two-dimensional correlation spectroscopy

The samples were prepared as a mixture of 1 mg of freeze-dried FA or of the FA-Al complex and 100 mg of potassium bromide (KBr, IR grade), and this mixture was then ground and homogenized [9], [10]. A subsample was then compressed twice in a hydraulic press at 20,000 psi between two clean, polished iron anvils to form a KBr window. The FTIR spectra were obtained by collecting 200 scans with a Nicolet 370 FTIR spectrometer.

The 2DCOS spectra were produced according to the method of Noda and Ozaki [11]. In this study, the Al concentration was applied as an external perturbation, and a set of concentration-dependent FTIR spectra was obtained. For example, in an analytical spectrum where *I(x, t)*, the variable *x* is the index variable representing the FTIR spectra induced by the perturbation variable *t*. The *x* was used instead of the general notation in conventional 2D correlation equations based on the spectral index *v*. The analytical spectrum *I(x, t)* at *m* evenly spaced points in *t* (between *T min* and *T max*) can be represented as
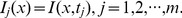
(1)


A set of dynamic spectra is given by
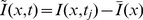
(2)where 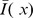 denotes the reference spectrum, which is typically the average spectrum that is expressed as 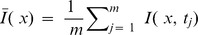. The synchronous correlation intensity can be directly calculated from the following dynamic spectra:



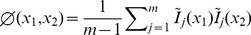
(3)The intensity of a synchronous correlation spectrum (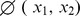) represents the simultaneous changes in two spectral intensities measured at *x_1_* and *x_2_* during the interval between *T_min_* and *T_max_*.

Prior to 2DCOS analysis, the FTIR spectra were normalized by summing the absorbance from 4000-400 cm^−1^ and multiplying by 1000. Subsequently, the normalized FTIR spectra were analyzed using principal component analysis (PCA) to reduce the level of noise [23]. Finally, 2DCOS was conducted using 2D shige software (Kwansei-Gakuin University, Japan).

In this study, we focused on the FTIR regions from 3600 cm^−1^ to 3100 cm^−1^, 1800 cm^−1^ to 900 cm^−1^, and 900 cm^−1^ to 400 cm^−1^ because they contain the major excitation bands of OH bonds, amides, carboxylic acids, esters, carbohydrates, Al-O and Si-O, and aliphatic C-H stretching [24], [25].

### Chemical analysis

The carbon (C) contents in the soil were analyzed using a Perkin-Elmer 2400 CHN elemental analyzer. The filtered (0.45 µm PTFE filter) extract was measured for DOC using a TOC/TN analyzer (multi N/C 3000, Analytik Jena AG, Germany). The pH was determined in 1∶2.5 (w/v) soil:water extracts using a pH electrode. Electrical conductivity (EC) was determined using a conductivity meter (LF91, German). The Al and Si concentrations in the soil FA extracts were measured using ICP-AES (Optima 7000, PerkinElmer, USA). Mineralogical identification was performed using X-ray powder diffraction (Nanjing Normal University). The diffraction patterns were recorded from 3 to 60° 2-theta using Ni-filtered Cu K-alpha radiation at a rate of 300 s per step on a PANalytical X-pert Pro diffractometer using an X-celerator position-sensitive detector.

### Statistical analysis

The data (means ± SD, *n* = 3) were assessed via one-way analysis of variance (ANOVA) using SPSS software version 18.0 for Windows (SPSS, Chicago, IL). Significance was determined using one-way ANOVA followed by Tukey’s HSD post hoc tests, where conditions of normality and homogeneity of variance were met, and means followed by different letters indicated significant differences between treatments at *P*<0.05.

## Results and Discussion

### Characterization of soil FAs in long-term fertilization treatments

As one of the main components of SOC, the soil FAs in the SM plot after 22 years of fertilization treatment contained a higher DOC concentration and EC than the FAs in the NPK plot (*p*<0.01, Table 1), which was consistent with the results that also showed that the SOC concentrations in the SM plot were significantly (*p*<0.01) greater (∼54±5%) than those in the NPK plot (Table 1). Therefore, consistent with previous publications, organic fertilization could markedly increase SOC content and soil fertility [26], [27]. Moreover, organic fertilization significantly increased (*p*<0.01) the concentration of weathered Al and Si and incorporated them into the soil FAs.

**Table 1:** Physiochemical characteristics of soil fulvic acids in the NPK and M treatments*.

Treatments	SOC	DOC (mg/L)	EC (m S/cm)	Al	Si
NPK	10.67±0.24c	570.6±11.8b	0.69±0.11b	3.3±0.2b	0.3±0.1b
SM	15.01±0.47a	893.3±23.5a	1.93±0.24a	6.8±0.5a	0.9±0.2a

*Note: Significance was determined using one-way ANOVA followed by Tukey’s HSD post hoc tests, where conditions of normality and homogeneity of variance were met, means (*n* = 3) followed by different letters indicate significant differences between treatments at *P*<0.05. Abbreviations: NPK, chemical nitrogen, phosphorus and potassium; SM, swine manure; SOC, soil organic carbon; DOC, dissolved organic carbon; EC, electricity conductivity.

**Figure 1:**
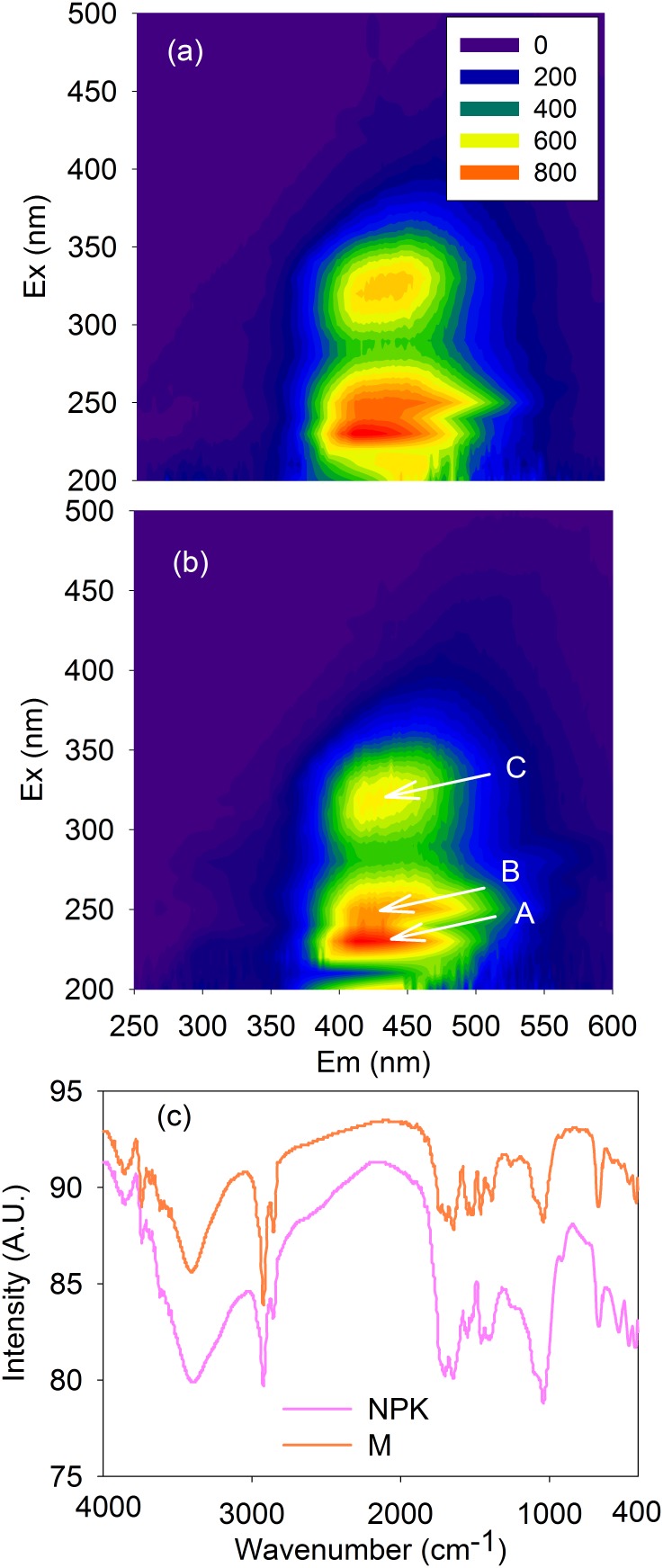
Fluorescence excitation-emission matrix (EEM) spectra of soil fulvic acids from the NPK (a) and SM (b) plots and FTIR spectra of the fulvic acids (c) during a long-term (i.e., 22-year) fertilization experiment. Note that peaks A, B and C were located at Ex/Em of 230/410, 250/410, and 330/410, respectively. NPK, chemical nitrogen, phosphorus and potassium; SM, swine manure.

Fluorescence EEM spectra demonstrated that three fluorescence peaks, i.e., peaks A, B, and C, which were located at Ex/Em of 230/420, 250/420, and 330/420, respectively, were present in the soil FAs from both the NPK and SM plots (Figures 1-a and 1-b) and were attributed to fulvic-like (i.e., peaks A and B) and humic-like (i.e., peak C) substances, respectively [8], [18], [28]. Although the fulvic-like (i.e., peaks A and B) substances were dominant, a considerable fraction of humic-like (i.e., peak C) substances was also present in the extracted soil FAs, suggesting that the soil FAs extracted by the operationally defined method actually include both FAs and HAs as indicated by fluorescence spectroscopy.

The organic ligands of the soil FAs from the NPK and SM plots were quite similar in the FTIR functional region (i.e., 4000-1000 cm^−1^), including OH from polysaccharides (3400 cm^−1^), CH_2_ from lipids (2920 cm^−1^), C = O stretching of the amide groups in proteins (1650 cm^−1^), C-N stretching and N-H bending vibrations of the amide groups in proteins (1540 cm^−1^), and Si-O (1020 cm^−1^) and Al-O-Si bands (610 cm^−1^ and 720 cm^−1^) in allophanes. However, the lignads were distinctly different in the fingerprint region (1000-400 cm^−1^) (Figure 1-c). In the fingerprint region, peaks at 720 cm^−1^ and 610 cm^−1^ were only present in the SM plot, both of them belonging to the Al–O–Si band in Al nanominerals [29], [30]. Different from the fluorescence EEM spectra, which report information only about fluorescence substances (i.e., protein- and humic-like substances), FTIR spectroscopy provides information about non-fluorescent substances (e.g., polysaccharides and lipids) and nanominerals (e.g., allophane) present in the soil FAs.

In summary, the long-term application of different fertilizers onto soils had little effect on the composition of fluorescent substances and organic ligands. The results were similar to those of a study by Randall et al. [31], in which mineral fertilizer and manure differed little in their effects on the chemical composition of SOM. Similar FTIR band characteristics were also previously reported between soil HAs in soils that were fertilized with manure and inorganic N fertilizer and an unfertilized control [32]. The minor effect of fertilization on the structures of the soil FAs may have been because the chemical composition of SOM is determined primarily by the interaction between the organisms responsible for decomposition and the mineral soil matrix rather than by the nature of the substrate input [31].

### Binding of organic ligands and nanominerals with Al in soil FAs

**Figure 2:**
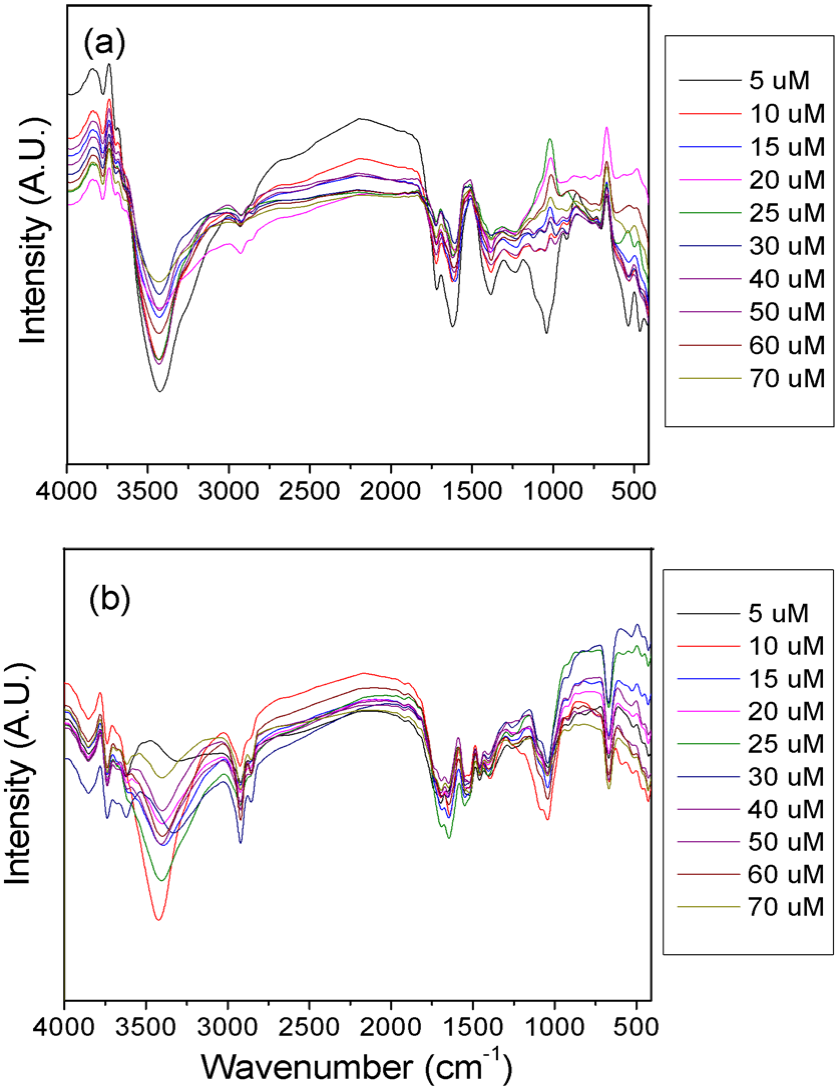
One-dimensional FTIR spectra of soil FAs from the NPK (a) and M (b) treatments during a long-term (i.e., 22-year) fertilization experiment. NPK, chemical nitrogen, phosphorus and potassium; SM, swine manure.

A better understanding of the binding of organic ligands and minerals to cations is essential to the stability of the SOC. Following the addition of Al, the major IR peaks appeared deformed in the one-dimensional FTIR spectra (Figure 2). The peaks were significantly overlapped, and no further information could be extracted. Therefore, two-dimensional correlation spectroscopy analysis was applied in this study to further analyze the binding of the organic ligands and minerals in the soil FAs to Al.

**Figure 3:**
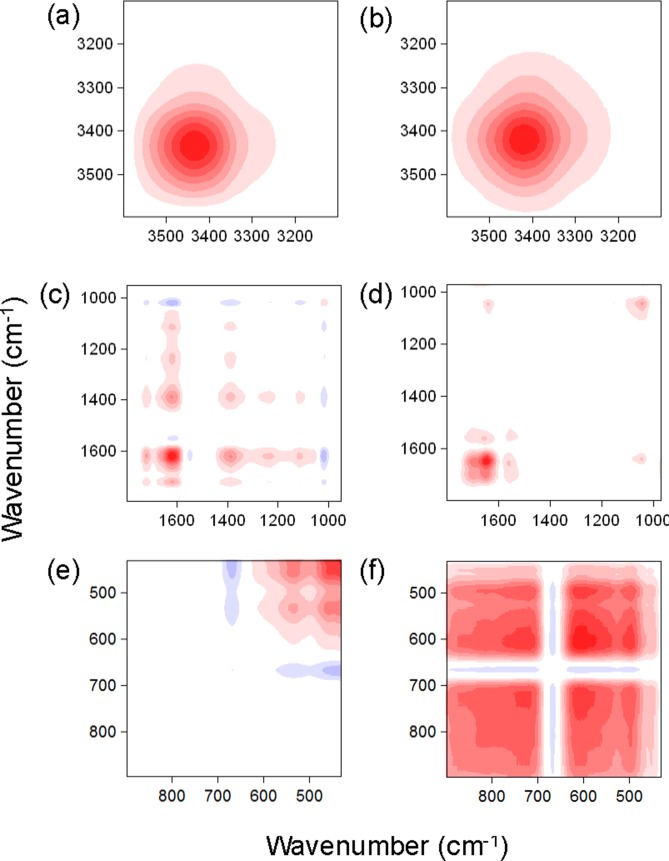
Synchronous 2D correlation maps generated from the FTIR spectra of soil FAs in the NPK (a, c and e) and SM (b, d and f) treatments with Al. Red represents positive correlation, and blue represents negative correlation; a higher color intensity indicates a stronger positive or negative correlation. NPK, chemical nitrogen, phosphorus and potassium; SM, swine manure.

Synchronous 2DCOS spectra of samples from the two plots are shown in Figure 3. In the 3600-3100 cm^−1^ region, only one auto-peak located at 3420 cm^−1^ (OH stretching in polysaccharides) was observed in both soil FAs from the two plots. In the 1800-900 cm^−1^ region, four and three major auto-peaks were present in the samples from the NPK and SM plots, respectively. The changes in band intensity decreased in the following orders: for the NPK plot, C = O stretching of amide I (1630 cm^−1^) > CH deformations in aliphatic groups (1380 cm^−1^) >> C = O stretching of COOH (1720 cm^−1^) ≈Si-O asymmetric stretching (1020 cm^−1^); for the SM plot, C = O stretching of amide I (1650 cm^−1^) > Si-O asymmetric stretching (1020 cm^−1^) >> C = N stretching of amide II (1540 cm^−1^). In the fingerprint region (900-400 cm^−1^), two and three auto-peaks were present in the NPK and SM plots, respectively. The band intensities decreased in the following orders: for the NPK plot, Si-O bending vibrations (430 cm^−1^) > Al-O-Si deformation (540 cm^−1^); for the SM plot, coupled Al-O and Si-O (out-of-plane) (610 cm^−1^) > Si-O stretching (720 cm^−1^) > Al-O-Si deformation (510 cm^−1^).

Seven positive cross-peaks at Φ(1630, 1380), Φ(1630, 1220), Φ(1630, 1080), Φ(1720, 1630), Φ(1720, 1380), Φ(1380, 1220), and Φ(1380, 1080) were present in the NPK fertilization, indicating that the C = O stretching of amide I, the CH deformations in the aliphatic groups, and the OH deformation of COOH were co-binding to Al. Moreover, four negative cross-peaks at Φ(1630, 1540), Φ(1630, 1010), Φ(1380, 1010), and Φ(1080, 1010) were also identified in the NPK fertilization, suggesting that the C = O stretching was negatively correlated with N–H deformations in the plane of amide II and that the Si-O stretching of the nanominerals was negatively correlated with C = O stretching, OH deformation of COOH, and CH deformations. In contrast, only two positive cross-peaks at Φ(1650, 1540) and Φ(1650 1020) were present in the SM fertilization, revealing that the C = O stretching of the amide groups were co-bound to Al, with N-H deformations of amide II and Si-O stretching of the nanominerals. In the fingerprint region, the presence of positive cross-peaks at Φ(540, 430) in the NPK plot and both Φ(720, 610) and Φ(720, 510) in the SM plot indicated that Si-O and Al-O-Si were co-bound to Al.

Although the long-term application of different fertilizers to soils had little effect with regard to organic ligands, this study showed, for the first time, that the different fertilization treatments had a marked effect on the binding of FAs with Al. First, more organic ligands in the 1800-900 cm^−1^ region were bound to Al in the NPK plot than in SM (Figures 3-b and 3-d). In contrast, except for the binding of organic ligands in the soil FAs with Al, the Si-O content (i.e., 1020, 720, and 610 cm^−1^) in minerals in the SM plot played a more important role in the binding to Al than in the NPK plot. This result was consistent with the finding of Yu et al. [8], in which a large amount of silicates was present in an NPK plus manure (NPKM) plot when compared to an NPK plot, suggesting that Al was bound to a silicate network in the SM plot. In addition to the binding of Al to the H-bond network (i.e., 3400 cm^−1^) (Figures 3-a and 3-b), it is reasonable to surmise that a large amount of allophone [Al_2_O_3_(SiO_2_)_1–2_(H_2_O)_2–4_] and/or imogolite [(OH)_3_Al_2_O_3_SiOH] was formed in the soil FAs in the M plot in contrast to the NPK plot.

### Morphology, diffraction pattern, and X-ray diffraction spectra of soil FAs

**Figure 4:**
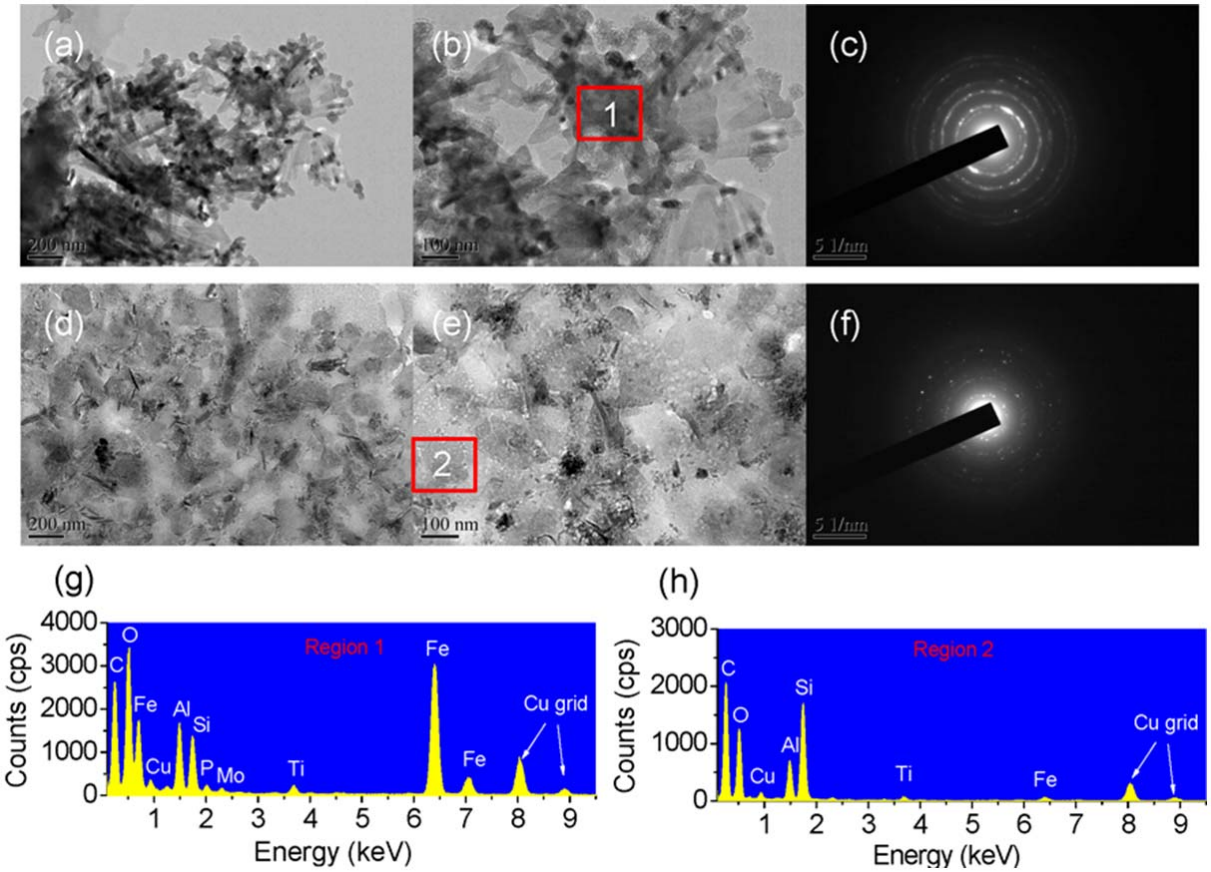
TEM images of the soil FAs from the NPK (a–b) and SM (d–e) treatments during a long-term (i.e., 22-year) fertilization experiment. Selected area electron diffraction (SAED) patterns of the two images indicated by the blue squares, demonstrate that the black regions are polycrystal (c) or short-ranged-order (SRO) (f) minerals. NPK, chemical nitrogen, phosphorus and potassium; SM, swine manure. (g) and (h) energy dispersive X-ray spectroscopy (EDS) of region 1 and 2 located at the red square frames.

**Figure 5:**
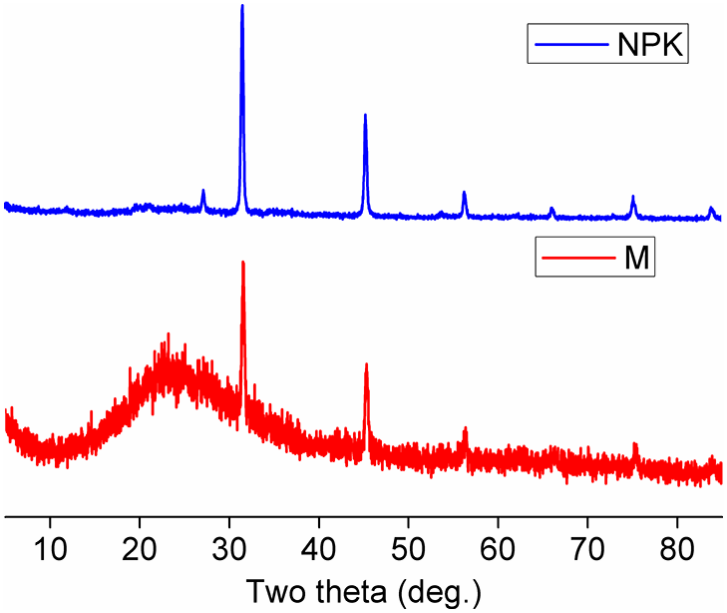
The X-ray diffraction pattern of soil FAs from the NPK and SM treatments during a long-term (i.e., 22-year) fertilization experiment. NPK, chemical nitrogen, phosphorus and potassium; SM, swine manure.

To confirm whether nanominerals were formed in the soil FAs in the SM and the NPK plots, TEM images and XRD spectra were used to observe the morphology and mineralogy of the nanominerals. A large amount of nanominerals was present in the TEM images of the soil FAs from both NPK (Figures 4-a and 4-b) and SM plots (Figures 4-d and 4-e). The electron diffraction patterns indicated that, although the soil FAs from both NPK and SM plots had a determinate crystalline and amorphous pattern, the obtained nanominerals were more crystalline under NPK conditions (Figure 4-c) than under SM conditions (Figure 4-f). Furthermore, the XRD spectra of the soil FAs corroborated that crystalline minerals were dominant under NPK fertilization conditions, while amorphous minerals were predominant under SM fertilization conditions (Figure 5).

Collectively, the TEM images and XRD spectra showed that nanominerals, i.e., allophone [Al_2_O_3_(SiO_2_)_1–2_(H_2_O)_2–4_] and/or imogolite [(OH)_3_Al_2_O_3_SiOH], were found abundantly in the soil FAs from the SM plot rather than from the NPK plot. In combination with the results of the 2DCOS spectra, the TEM images and XRD spectra showed that the nanominerals in soil FAs may play an important role in the binding of metals, which should benefit both soil remediation and soil C sequestration.

### Implications to soil organic carbon sequestration

The results from the 2D FTIR correlation spectroscopy, TEM images and XRD spectra point to the formation of soil nanominerals in the SM rather than in the NPK treatments. The formation of soil nanominerals (i.e., allophone and imogolite) could greatly benefit the preservation of SOC [33]. Torn et al. [34] previously demonstrated that soil nanominerals controlled SOC storage and turnover. Under favorable conditions, the turnover of SOC in allophonic soils could persist in tephra beds for at least 250,000 years [7]. Globally, approximately 190–332 million tons of C can be sequestered by silicate minerals annually [35]. As one of the main components of SOC, the abundance of nanominerals in the soil FAs from the SM plot would definitely contribute to the stability of the SOC. The knowledge of the binding of organics and metals improves our understanding of the sequestration process of SOC and provides valuable information with regard to fertilization techniques and scientific research.

## Conclusions

In this study, FTIR spectroscopy combined with 2DCOS analysis has been developed to investigate the binding capability between soil FA and mineral elements.

The results show that the organic ligands in the soil FAs were mainly bound to Al in the NPK treatment, whereas both organic ligands and minerals (Al–O–Si, Si–O) were bound to Al in the SM treatment. The abundance of amorphous and short-range-ordered (SRO) nanominerals in the soil FAs of the SM plot, rather than the NPK plot, illustrated the preservation role of the soil FAs by nanominerals during long-term organic fertilization. In summary, the combination of FTIR and 2D correlation spectroscopy is a promising approach for the characterization of the binding capability between soil FA and mineral elements. A better understanding of this binding capability will benefit the understanding of soil C sequestration and greatly contribute to global C cycling.
